# Investigation on Cell Disruption Techniques and Supercritical Carbon Dioxide Extraction of *Mortierella alpina* Lipid

**DOI:** 10.3390/foods11040582

**Published:** 2022-02-17

**Authors:** Chang Chng Ong, Yen-Hui Chen

**Affiliations:** Department of Food Science, National Ilan University, No. 1, Sec. 1, Shennong Rd., Yilan City 260, Taiwan; eric-ong93@hotmail.com

**Keywords:** *Mortierella alpina*, cell disruption, supercritical carbon dioxide, arachidonic acid, lipid

## Abstract

*Mortierella alpina*, an oleaginous fungus, has been shown to be a potential source for arachidonic acid (ARA) production. The recovery of intracellular lipids from *M. alpina* is an important step for the downstream bioprocessing, and green extraction techniques with a focus on being efficient and eco-friendly have drawn much attention. In this study, different cell disruption techniques (mechanical: high-speed homogenization 10,000 rpm, ultrasonication 20 kHz, high-pressure process (HPP) 200–600 MPa; non- mechanical: acid treatment HCl) were investigated for lipid recovery from *M. alpina*, and process parameters (A. temperature, B. pressure, C. cosolvent ratio) of supercritical carbon dioxide (SC-CO_2_) lipid extraction were studied by applying response surface methodology (RSM). Compared with Soxhlet extraction as a control group (100%), high-speed homogenization has the highest lipid recovery (115.40%) among mechanical disruption techniques. Besides, there was no significant difference between high-speed homogenization and 1 M HCl treatment (115.55%) in lipid recovery. However, lipid recovery decreased to 107.36% as the concentration of acid was increased to 3 M, and acid treatment showed a negative effect on the ARA ratio. In HPP treatment, the highest lipid recovery (104.81%) was obtained at 400 MPa, 1 time of treatment and water medium. In the response surface model of SC-CO_2_ extraction, results showed the major influence of the process parameters to lipid recovery was pressure, and there are interaction effects of AC (temperature and cosolvent ratio) and BC (pressure and cosolvent ratio). Lipid recovery of SC-CO_2_ extraction reached 92.86% at 201 bar, 58.9 °C and cosolvent ratio 1:15. The microbial lipid recovery process of this study could be used as a reference and an eco-friendly alternative for the future downstream bioprocessing of ARA production by *M. alpina*.

## 1. Introduction

The filamentous fungi *Mortierella alpina* is a potential source of arachidonic acid (ARA). The conventional source of ARA is mainly from deep-sea fish, animal tissues (adrenal gland and liver) and egg yolk [1]. However, the separation and purification costs are high, and the production could not adequately meet the ever-increasing demand in pharmaceutical and nutraceutical industries. Microorganisms, such as algae and filamentous fungi, are major alternative sources for ARA production [2]; however, algae exhibited relatively low productivity of ARA (0.1–0.2 g/L-d), and *M. alpina* is currently considered as a viable alternative which is employed in the industrial production due to its high ARA productivity (0.5–2.8 g/L-d) [1,3,4].

ARA, an important polyunsaturated fatty acid (PUFA) of ω-6 series, acts as a precursor of bioactive eicosanoids such as prostaglandin, thromboxane and leukotriene via the catalysis of cyclooxygenase and lipoxygenase. These active substances have important physiological effects for maintaining lipid metabolism, smooth muscle contraction and enhancing immune cell function [5]. Due to the versatility of ARA, it has a wide range of applications in food, medical care, cosmetics and other fields. In addition, ARA is an essential fatty acid for infants, which has an important influence on the development of the infant’s brain, nervous system and vision [6].

ARA is an intracellular product of *M. alpina*, and with a proper cell disruption could help to improve extraction efficiency. Conventional cell disruption of the oleaginous microorganism can be classified into mechanical and non-mechanical methods. Mechanical methods include autoclaving, microwaving, ultrasonication, bead milling, high-pressure homogenization and high-speed homogenization, while non-mechanical include acid and alkali treatment, enzyme treatment and osmotic shock [7]. The variables that influence the cell disruption in the oleaginous microorganism should be studied, aiming for greater lipid recovery and the quality of the extracted material. To the best of our knowledge, using different cell disruptions, such as chemical, enzymatic, ultrasonic, microwaving, autoclaving, electroflotation and bead-beating on microalgae or yeast are largely investigated [8,9,10,11,12], but few studies exist on *M. alpina* [13,14,15]. Enzymatic treatment was found suitable for extracting carotenoids from *Sporidiobolus salmonicolor* [8], astaxanthin from *Haematococcus pluvialis* [9] and lipids from *M. alpina* [13,14]. Acid digestion was proposed as a simple and effective method for lipid recovery from fungus *Mortierlla isabellina*, yeast *Cryptococcus curvatu* and microalga *Chlorella sorkiniana* [11].

In the downstream bioprocess, an effective cell disruption/assisted extraction method is critical for intercellular microbial lipid recovery, and green-eco and sustainable technology, such as supercritical carbon dioxide (SC-CO_2_) and high-pressure processes (HPP) have drawn much attention [12,16]. HPP, also called high-hydrostatic pressure, as an alternative to heat decontamination methods has shown the effect on cell membrane integrity and molecular composition [17], and it might be used for improving extraction efficiency [16,18]. The advantages of applying HPP include the preservation of nutrients, colors, uniformity of treatment throughout, shorter processing time and potential for a reduction in chemical preservatives [19]. Moreover, organic solvent extraction of microbial lipids is normally considered a developed technology, and research is in progress to investigate alternative solvents that are safer than hexane and moderate for the environment. SC-CO_2_ is a promising green solvent that may potentially substitute the use of organic solvents for lipid extraction that offers the advantages of non-explosive, non-toxic, non-flammable and readily available. The complete removal of normally gaseous solvent from final products is another major advantage compared to liquid solvents that often leave toxic residues [20]. The efficiency of SC-CO_2_ extraction may be affected by many variables, including temperature, pressure, extraction time, CO_2_ flow rate and particle size, individually or in combination [21]. For instance, the change of operation temperature and/or pressure can alter the CO_2_ density, thereby manipulating the solvent power of CO_2_ [22,23].

The objectives of this project were to investigate different cell disruption techniques (high-speed homogenization, ultrasonication, HPP and acid treatment) for *M. alpina* lipid recovery and to study the model of SC-CO_2_ extraction for discussing the effectiveness of process parameters (temperature, pressure and cosolvent ratio). 

## 2. Materials and Methods

### 2.1. Microorganism Cultivation

*M. alpina* FU30797 was obtained from Bioresource Collection and Research Center (BCRC, Taiwan). It was maintained on potato dextrose agar (PDA) plates and transferred every two weeks. 3 × 1 cm^2^ of culture grown on PDA plate was inoculated into 200 mL seed medium containing (g/L): glucose 20.0, yeast extract 30.0, KH_2_PO_4_ 0.2 incubated for 48 h at 25 °C under constant orbital shaking at 200 rpm. A 48 h cultivated pallet (200 mL) was homogenized with HG-300D homogenizer for 10 s at 100 rpm. 200 mL (10%, *v*/*v*) mycelial suspension was inoculated to 1.8 L fermentation media containing (g/L): glucose 40.0, yeast extract 8.0, KH_2_PO_4_ 0.2, MgSO_4_ 0.5 and incubated at 25 °C, 200 rpm, aeration 3.0 L/min for 120 h. After cultivation, the culture medium was harvested at 10,000× *g* during 15 min to separate the fungi cell, which was washed twice and then freeze dried for further experiments.

### 2.2. Cell Disruption—Assisted Extraction Techniques

#### 2.2.1. High-Speed Homogenizer

High-speed homogenizer procedure was modified from a previous study [24]. In total, a 0.5 g sample was extracted in a 50 mL centrifuge tube using 5 mL n-hexane, homogenized with HG-300D homogenizer for 60 s at 10,000 rpm and centrifuged at 3000× *g* for 10 min. The extraction was repeated twice. Supernatant was combined, transfer to another tube through 0.45 μm syringe filter and evaporated by using nitrogen. The dried biomass and lipids were measured by analytical balance.

#### 2.2.2. Acid Treatment

Acid procedure was modified from a previous study [12]. In total, a 0.5 g sample was added to 5 mL of hydrochloric acid (HCl) solution at different concentrations (1, 2 and 3 M) maintained in a water bath at 35 °C, 100 rpm for 15 min. The residue was rinsed with RO water on filter paper and then dried at 25 °C, and Soxhlet extraction method with hexane as the solvent was applied for further lipid extraction (Section 2.4).

#### 2.2.3. Ultrasonication

A 0.5 g sample was added to 10 mL n-hexane in a test tube, and then it was immersed in a standard 1/2” ultrasonic probe connected to an ultrasound transducer (model CL5) with power control (S-4000, Misonix Inc., Farmingdale, NY, USA). The output frequency was 20 KHz and ultrasonic condition was set to 20 W for 30 s [12]. The residue was treated as Section 2.2.2.

#### 2.2.4. High-Pressure Processing (HPP)

A 0.5 g sample was added to 10 mL solution (water or 95% ethanol) in the retort pouch (130 × 210 mm) and then sealed. The experimental pressure was set at 200, 400 and 600 MPa, the number of treatments was set for once or twice (each for 2 min). The volume of the HPP instrument was 6.2L (HPP600MPa, Kuentai International Co., Ltd., Yunlin, Taiwan). The residue was treated as Section 2.2.2. 

### 2.3. Supercritical Carbon Dioxide (SC-CO_2_) Extraction

The SC-CO_2_ extraction experiment was modified from a previous study [10], and each experiment was carried out with an extraction apparatus (OV-SCF-10000C, Taiwan Supercritical Technology, Changhua, Taiwan) which consisted of a 100 mL extraction vessel, a pump, a temperature-controlled oven and sample collection. For each experiment, 0.5 g of biomass was mixed with glass beads (3 mm diameter), and glass wool was packed into the top and bottom of the vessel to avoid the entrainment of biomass, and stainless-steel frits were coupled at both ends of the vessel to evenly distribute the SC-CO_2_ fluid. For establishing a suitable extraction condition, RSM with a three-factors and three-levels Box–Behnken design was employed and analyzed by using Design-Expert software. Lipid extraction was carried out according to the experimental design (coded level): temperature 40(−1), 50(0), 60(1) °C; pressure 200(−1), 250(0), 300(1) bar; ethanol cosolvent ratio 1:5(−1), 1:10(0), 1:15(1); with 17 runs and 5 center points. For each run, the equilibrant time was 40 min, and then dynamic extraction was applied for 30 min with a flow rate 6 L/min. The extracted lipid was gravimetrically quantified and analyzed for fatty acid composition.

### 2.4. Lipid Recovery Calculation

The lipid recovery percentage was calculated by the following equation:

(1) $$\mathrm{Lipid}\;\mathrm{Recovery}\;\%=\frac{\mathrm{Weight}\;\mathrm{of}\;\mathrm{extracted}\;\mathrm{lipid}\;\left(g\right)}{\mathrm{Weight}\;\mathrm{of}\;\mathrm{total}\;\mathrm{lipid}\;\left(g\right)}\times 100$$

The analytical procedure of the total lipid in the untreated *M. alpina* biomass was modified from Method of Test for Fatty Acids in Food: Fat extraction—MOHWO0014.00 (Food and Drug Administration, Taipei, Taiwan). The Soxhlet method was applied for lipid extraction, and it was carried out as follows: lipids were extracted from a 0.5 g sample in a Soxhlet extractor with hexane as the solvent at 95 °C for 8 h, and the extraction solution was evaporated to dryness under a vacuum at 40 °C for obtaining extracted lipids.

### 2.5. Fatty Acid Composition Analysis

Fatty acid composition of crude lipid was determined by using GC-FID (Clarus^®^ 680, Perkin Elmer, Waltham, MA, USA) and a 100 m HP-88 capillary column (0.25 mm i.d. and 0.20 µm film thickness). Column temperature was programmed to hold at 170 °C for 40 min, and then increase to 200 °C at 3 °C per min and held for 50 min. The injector and detector were maintained at 250 °C and 300 °C. Sample injection volume was 1.0 μL. Nitrogen was the carrier gas at a flow rate of 0.75 mL per min with a split ratio of 1:40. Peaks were identified by comparison to reference standards of 37 components FAME mix (Supelco, Bellefonte, PA, USA).

### 2.6. Scanning Electron Microscopy

The control (Soxhlet extraction) biomass and treated cells were examined by scanning electron microscopy (SEM) to investigate the morphological changes in fungal cells during the disruption process. For SEM analysis, the control, acid disruption and ultrasonication treated biomass were mounted on aluminum stubs and coated with a gold layer. Samples were scanned with Hitachi TM3000 scanning electron microscopy at an accelerating voltage of 5 kV.

### 2.7. Statistical Analysis

Statistical analysis was performed with SPSS 18.0 software. The extractions were carried out in triplicate and the mean values are reported. The comparison of the results from the control and different treatments was carried out by one-way analysis of variance (ANOVA) and post hoc analysis was performed by Duncan’s multiple range test. The values are given as mean ± SD. Levels of significance were considered α = 0.05.

## 3. Results and Discussion

### 3.1. Lipid Recovery of Different Cell Disruption

**Figure 1:**
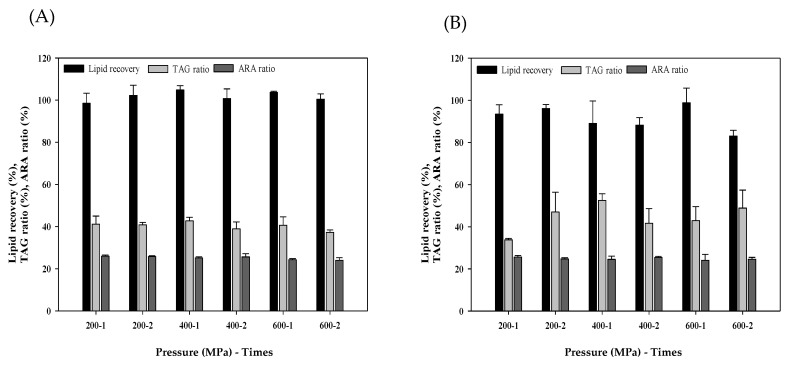
Lipid recovery, TAG ratio of dry matter and ARA ratio of total fatty acid by HPP treatment on *M. alpina* FU30797 by different pressure (MPa) and times of treatment. Different pressure medium (**A**) water, (**B**) ethanol. (*n* = 3).

The effect of different treatments on the lipid recovery of *M. alpina* FU30797 was shown in Table 1 and Figure 1. An untreated sample with Soxhlet solvent extraction was used as a control group to compare with the different cell disruption techniques. As shown in Table 1, high-speed homogenization breaks the cell wall with shear force and grinding with high-speed rotation; therefore, it will maximize the breaking of the cell wall, improving the lipid recovery significantly by obtaining the highest lipid recovery (115.40%). Ultrasonication treatment breaks the cell wall by shear force generated by cavitation effect, which has a slightly increased lipid recovery (101.73%), the reason might be due to weak shear strength insufficient to damage the cell wall. Similar results were found in the lipid recovery of mixed microalgae, de Souza Silva et al. reported total lipid yield of ultrasonic treatment was 13.3% which is the lowest compared with microwaving, autoclaving and electroflotation by alternating current [10].

By using acid treatment, the lipid recovery reached 115.55% in 1 M HCl, and there was no significant difference to that of high-speed homogenization. The acid treatment would change the permeability of the cell wall, which might make it easier for the solvent to extract the intracellular lipids. However, lipid recovery decreased to 107.36% as the concentration of acid was increased to 3 M, and acid treatment negatively affected the ARA ratio. Duarte et al., studying the different cell disruption techniques (HCl, hydrothermal hydrolysis, high-pressure homogenization, maceration, ultrasound and shear abrasion) to yeast *Candida* sp. LEB-M3, observed that lipid recovery was increased (106–107%) when yeast was treated by acidic medium (HCl 2–5 M) [12]. In addition, lipid recovery reached 155% by using hydrothermal hydrolysis in HCl 2 M medium (121 °C/101 kPa), and changes in the structure with some cell surface deformation was found when compared with the biomass before the technique application.

Enzymatic hydrolysis, one of other non-machinal cell disruption techniques, was also studied for the extraction of lipid from *M. alpina* [14]. Papain, pectinase, snailase, neutrase, alcalase and cellulase were applied, respectively. The highest enzyme efficiency was obtained in neutrase treatment, but the lipid recoveries were all below 50%; thus, You et al. further investigated the ratios of enzyme mixtures and the effects of enzyme concentration, temperature and hydrolysis time on lipid recovery. Results showed the lipid recovery reached 101.3% when pectinase and alcalase (5:1, *v*/*v*) was used, and the highest lipid recovery was obtained by using pectinase and papain (5:3, *v*/*v*). Moreover, the enzymatic treatment would not affect the fatty acid composition. Nisha et al. reported applying *Trichoderma viride* lytic enzyme could obtain better lipid recovery (51.47%) of *M. alpina* (CBS 528.72) compared with untreated biomass [13]. Moreover, Wang et al. reported the combination of enzymatic (1% papain and 1% cellulase) and mechanical cell disruption (ultrasonication and high-pressure homogenization) for lipid extraction from green alga *Neochloris oleoabundans*. In the conformation of enzyme and high-pressure homogenization, the highest disruption was obtained (up to 95.41%), and the lipid recovery reached 92.6% [25].

HPP is a non-thermal processing technology for heat sensitive products, and the common pressure range of HPP is about 100–1000 MPa. In the HPP experiment, the highest lipid recovery (104.81%) was obtained at 400 MPa, 1 time and water medium, and the ARA and TGA were not affected much while increasing the pressure and treatment (Figure 1A). HPP has shown the effect on cell membrane integrity and molecular composition [17], electrostatic disruption and hydrophobic interactions in food protein [26]. In a different pressure medium, the overall lipid recovery of ethanol was lower than that of water, because the lipid was partially soluble in ethanol, but the highest TAG ratio (52.52% of dry matter) was found at 400 MPa, 1 time and ethanol medium. In addition, the higher lipid recovery was obtained in 600 MPa, 1 time in the ethanol group. Nevertheless, lipid loss may occur along the procedure including drying, packaging and filtering, which caused less lipid recovery. To the best of the authors’ knowledge, no published article has investigated HPP treatment on *M. alpina* biomass.

Reboleira et al. had studied high-hydrostatic pressure treatment for *Aurantiochytrium* sp., an alternative source of PUFAs-DHA and squalene, lipid extraction [16]. Three different pressure media (ethanol, ethyl acetate and hexane), pressure (500 MPa) and time (15 min) were applied, and lipid was recovered directly from pressure media. Results found the lipid recovery of ethanol treatment was about 10% higher than that of Soxhlet, and ethanolic high-hydrostatic pressure extracts had the inhibition effect of lipid oxidation reactions and microbial growth. However, the lipid yield of hexane treatment was increased to approximately two times as compared with that of ethanol group, and it might due to better lipid solubility of hexane. We both found the potential of HPP for increasing the efficiency of lipid extraction from a microorganism. Besides, Yusoff et al. found HPP in changing the protein structure of *Moringa oleifera* into a form of less emulsifying functional properties; therefore, a further de-emulsification process might not be required [27], and it showed HPP pretreatment might improve the efficiency of the lipid extraction processing.

### 3.2. SEM Image of M. alpina after Different Cell Disruption

**Figure 2:**
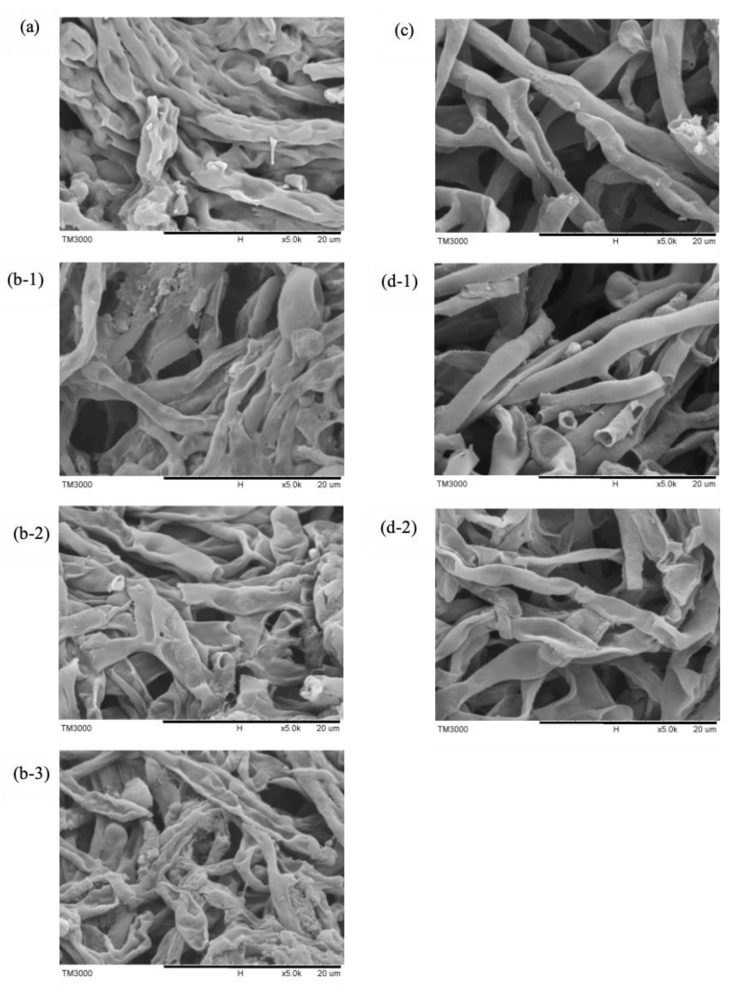
SEM images of *M. alpina* FU30797 in 5k magnitudes, where: (**a**) untreated biomass, (**b**) HCl treatment: 1 M (**b-1**), 2 M (**b-2**), 3 M(**b-3**), (**c**) ultrasonication 20 W and (**d**) HPP (600 MPa, 1 time) by water medium(**d-1**) and ethanol medium(**d-2**).

The fungal cell wall is rigid which might be higher than that of a plant cell [13], and the resistance of the cell wall could affect the rate of mass transfer, indicating a critical step for lipid extraction from a fungal cell. Figure 2 shows the images obtained by scanning electron microscopy (SEM) of *M. alpina* FU30797 in 5k magnitudes, before and after the application of the cell disruption.

The images of *M. alpina* FU30797 treated with chemical HCl (Figure 2b) revealed a greater rupture of the cell wall in the treated cells compared to that of the intact cell (Figure 2a), resulting in higher lipid recovery of acid treatment. When the higher concentration of acid treatment was applied, the greater the deformation of the cell structure and small cell fragments could be observed; however, the maximum lipid recovery (115.55%) was obtained in HCl 1 M treatment, and the minimum ARA ratio was found in HCl 3 M treatment. Nisha et al. used HCl 0.1 N to treat *M. alpina* (CBS 528.72) biomass, and the SEM image also indicated cell deformation [13]. In contrast, Durate et al. reported lipid recovery of yeast *Candida* sp. LEB-M3 was increased with a higher concentration of HCl treatment up to 5 M, and the chemical reaction probably caused by the acid improved the breaking of bonds in the cell structure [12]. These indicated that a suitable concentration of HCl treatment for different strains might be different. Within the concentration range (1–3 M) studied in this work, HCl 1 M treatment was considered as a proper chemical treatment for lipid recovery and ARA ratio.

The low efficiency of ultrasonication treatment is confirmed by the image of cells after the application of ultrasonic waves as shown in Figure 2c. Due to weak shear strength, slight damage was observed in the cell wall structure when compared with the biomass image obtained before cell disruption, contributing to lower lipid recovery (Table 1) in comparison with other cell disruption techniques. Wang et al. obtained the same SEM results by extracting algae *Neochloris oleoabundans* with ultrasonication (600–800 W), and lipid recovery was lower than high-pressure homogenization (60–80 MPa) because the cell was fragmented completely after homogenization.

As shown in Figure 2d, HPP didn’t cause the cell rupture of *M. alpina* FU30797 significantly, but lipid recovery was slightly higher than that of the treatment at 400 MPa, 1 time and water medium, and it might be because the effect of HPP was on molecular composition and cell membrane integrity [17], which probably requires higher magnitudes to observe the difference. Besides, the shrinkage was found in the ethanol group (Figure 2d-2. The effect of HPP for lipid extraction from *Aurantiochytrium* sp. was proved [16], and a longer application time and suitable solvent was suggested for further lipid recovery from *M. alpina* FU30797.

### 3.3. Supercritical Carbon Dioxides (SC-CO_2_) Extraction of M. alpina Lipid

Enzyme and HCl pretreatment could improve lipid recovery of *M. alpina* (CBS 528.72) were reported [13], but efficiency of lipid extraction by applying SC-CO_2_ is lower than that by using Sox Tech^TM^ method. In our preliminary experiment (250 bar, 50 °C and cosolvent 1:10), HCl 1 M pretreatment was performed, but lipid recovery of *M. alpina* FU30797 was only 83.1%, and it is speculated that a high concentration of acid would cause the shrinkage and damage of the cell wall, which might affect the penetration of SC-CO_2_ solvent. Furthermore, the efficiency of SC-CO_2_ extraction may be affected by many variables, including temperature, pressure, extraction time, CO_2_ flow rate, cosolvent and particle size individually or in combination [21]. For instance, the change in operation temperature and/or pressure can alter the CO_2_ density and diffusibility, thereby manipulating the solvent power of CO_2_. In this study, the influence of pressure, temperature and the ratio of ethanol as a cosolvent were investigated by applying RSM with Box–Behnken design, and untreated biomass was chosen as the samples. As shown in Table 2, the analysis of the response surface two-factors-interaction model was significant (*p* < 0.05), and the lack of fit was not significant (*p* > 0.05). In addition, the major influence of the experiment factors on lipid recovery was pressure, and AC (temperature and cosolvent ratio) and BC (pressure and cosolvent ratio) had the effect of interaction. The prediction model in termed of coded factors is: lipid recovery = 89.64 + (1.07 × A) + (2.83 × B) + (1.69 × C) + (1.42 × A × B) + (4.92 × A × C) − (5.51 × B × C). Suitable lipid recovery was 92.86% at 201 bar, 58.9 °C and cosolvent ratio 1:15 from triplicate experimental verification, and there was no significant difference between SC-CO_2_ and Soxhlet solvent extraction in ARA ratio.

**Figure 3:**
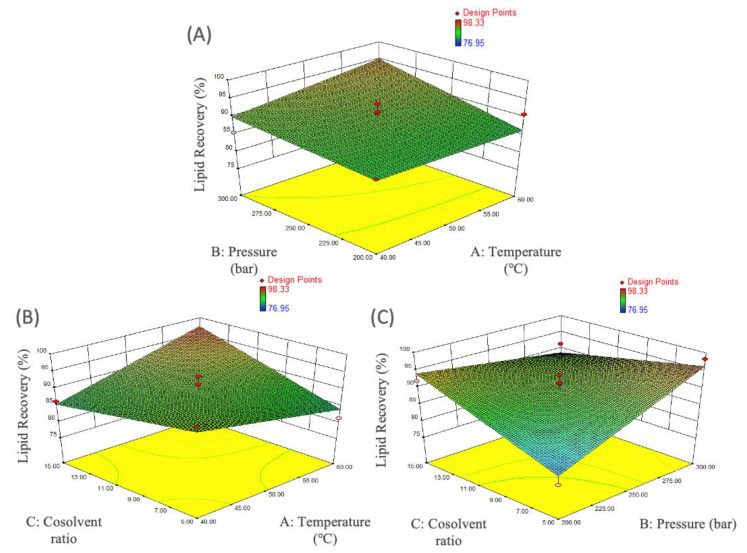
Response surface plot fixed on (**A**) cosolvent ratio 1:10, (**B**) pressure 250 bar and (**C**) temperature 50 °C to show the effect of pressure, temperature and cosolvent ratio on lipid recovery of *M. alpina* FU30797 from SC-CO_2_ extraction.

As shown in Figure 3A, lipid recovery had no significant changes as the pressure was increased in the low level of temperature (40–50 °C), but it was slightly raised as the pressure was increased in the low level of temperature (50–60 °C), and it reached highest around 56–60 °C and above 285 bar. In addition, lipid recovery was increased in high levels of temperature (50–60 °C) and cosolvent ratio (1:10–15) (as shown in Figure 3B). An increase in lipid recovery was observed with rising pressure at temperatures likely due to increased vapor pressure of solutes, SC-CO_2_ density and mass transfer rate, which resulted in the elevation of the lipid solubility in SC-CO_2_ [28]. Tanaka et al. reported that the extraction with ethanol as a cosolvent in SC-CO_2_ was effective in lipid recovery. Similar results were also published for SC-CO_2_ lipid extraction of heterotrophic microalga *Crypthecodinium cohnii* [29]. Moreover, there was a repulsing effect of low pressure (200–250 bar) and cosolvent ratio (1:5–10) condition due to lower solute solubility in this range (Figure 3C).

## 4. Conclusions

This work demonstrated different cell disruption techniques for the cell disruption of fungus *M. alpina* FU30797 by using convectional solvent extraction, and results indicated that the high-speed homogenization and HCl 1 M treatment could increase the lipid recovery of *M. alpina* FU30797 up to 15%. SEM images showed evidence of cell condition after disruption treatment. Moreover, analysis of the SC-CO_2_ extraction data using RSM explained the effects of processing parameters and provided a prediction model of lipid recovery. In addition, lipid recovery was over 90% without the pretreatment, indicating that SC-CO_2_ extraction could be competitive with the conventional process because it completely eliminates the solvent distillation stage; therefore, this microbial lipid recovery process presented a promising effective and eco-friendly alternative.

## Figures and Tables

**Table 1:** Lipid recovery of different disruption treatments applied in *M. alpina* FU30797 biomass.

Treatment	Lipid Recovery (%)	ARA Ratio (%)
High-speed homogenizer	115.40 ± 3.47 ^a^	31.72 ± 0.65 ^a^
Acid disruption (HCl)		
1 M	115.55 ± 3.88 ^a^	29.03 ± 0.19 ^b^
2 M	112.95 ± 0.31 ^a^	29.21 ± 0.18 ^b^
3 M	107.36 ± 1.47 ^b^	28.23 ± 0.19 ^c^
Ultrasonication	101.73 ± 4.44 ^b^	28.91 ± 0.55 ^bc^

Value represent Mean ± SD (*n* = 3). ^a–c^ Means with different superscript in the same column were significantly different (*p* < 0.05). Lipid recovery of each treatment was compared with Soxhlet extraction with untreated biomass as a control (100%), and there is no significant difference between the control (31.52 ± 0.56%) and high-speed homogenizer treatment in ARA ratio.

**Table 2:** ANOVA for response surface two-factors-interaction model for lipid recovery of *M. alpina* FU30797.

Source	Sum of Squares	df	Mean Square	F-Value	*p*-Value Prob > F
Model	322.36	6	53.73	4.89	0.0138
A: Temperature	9.10	1	9.10	0.83	0.3842
B: Pressure	64.24	1	64.24	5.85	0.0361
C: Cosolvent ratio	22.85	1	22.85	2.08	0.1797
AB	8.12	1	8.12	0.74	0.4099
AC	96.73	1	96.73	8.81	0.0141
BC	121.33	1	121.33	11.05	0.0077
Residual	109.80	10	10.98		
Lack of fit	84.36	6	14.06	2.21	0.2313
Pure error	25.45	4	6.36		
Cor total	432.17	16			

## Data Availability

Not applicable.
